# Effects of three-dimension movie visual fatigue on cognitive performance and brain activity

**DOI:** 10.3389/fnhum.2022.974406

**Published:** 2022-10-19

**Authors:** Ryota Akagi, Hiroki Sato, Tatsuya Hirayama, Kosuke Hirata, Masahiro Kokubu, Soichi Ando

**Affiliations:** ^1^College of Systems Engineering and Science, Shibaura Institute of Technology, Saitama, Japan; ^2^Graduate School of Engineering and Science, Shibaura Institute of Technology, Saitama, Japan; ^3^Faculty of Sport Sciences, Waseda University, Tokorozawa, Japan; ^4^Faculty of Health and Sport Sciences, University of Tsukuba, Tsukuba, Japan; ^5^Graduate School of Informatics and Engineering, The University of Electro-Communications, Chofu, Japan

**Keywords:** critical flicker fusion frequency (CFF), functional near-infrared spectroscopy (fNIRS), Go/NoGo task, visual task, auditory task

## Abstract

To further develop three-dimensional (3D) applications, it is important to elucidate the negative effects of 3D applications on the human body and mind. Thus, this study investigated differences in the effects of visual fatigue on cognition and brain activity using visual and auditory tasks induced by watching a 1-h movie in two dimensions (2D) and 3D. Eighteen young men participated in this study. Two conditions were randomly performed for each participant on different days, namely, watching the 1-h movie on television in 2D (control condition) and 3D (3D condition). Before and after watching the 1-h movie on television, critical flicker fusion frequency (CFF: an index of visual fatigue), and response accuracy and reaction time for the cognitive tasks were determined. Brain activity during the cognitive tasks was evaluated using a multi-channel near-infrared spectroscopy system. In contrast to the control condition, the decreased CFF, and the lengthened reaction time and the decreased activity around the right primary somatosensory cortex during Go/NoGo blocks in the visual task at post-viewing in the 3D condition were significant, with significant repeated measures correlations among them. Meanwhile, in the auditory task, the changes in cognitive performance and brain activity during the Go/NoGo blocks were not significant in the 3D condition. These results suggest that the failure or delay in the transmission of visual information to the primary somatosensory cortex due to visual fatigue induced by watching a 3D movie reduced the brain activity around the primary somatosensory cortex, resulting in poor cognitive performance for the visual task. This suggests that performing tasks that require visual information, such as running in the dark or driving a car, immediately after using a 3D application, may create unexpected risks in our lives. Thus, the findings of this study will help outlining precautions for the use of 3D applications.

## Introduction

Recently, there has been widespread use of three dimensional (3D) applications in a variety of situations such as education (Tilhou et al., 2020; Webb et al., 2022), healthcare (Chirico et al., 2016; O’Connor et al., 2021; Zawy Alsofy et al., 2021), and entertainment (Oriti et al., 2021; Peters et al., 2021). The use of 3D applications in education ought to be prioritized, particularly with the introduction of online classes as exacerbated by the COVID-19 pandemic (Ng, 2022; Shin et al., 2022; Vergara et al., 2022). Thus, it is essential to elucidate the negative effects of 3D applications on the human body and mind for the future development of 3D applications.

Visual fatigue is a major concern related to the use of 3D applications (Iatsun et al., 2015; Sugita et al., 2019). Chen et al. (2015) have described that the uncoupling of convergence and accommodation that reduces one’s ability to fuse the binocular stimulus predominantly causes visual fatigue induced by 3D viewing. Iatsun et al. (2015) investigated the number of saccades and fixations as well as their duration during a 1-h movie viewed either in two dimensions (2D) or 3D. Iatsun et al. (2015) found that the number of saccades, fixations, and fixation duration decreased with time in the 3D condition, whereas no change in any of the indices was observed over time in the 2D condition. In another study (Cho et al., 2017), the accommodative response and ocular movements revealed noticeable changes while viewing 3D movies (30 min) in contrast to 2D movies, and both the accommodation and binocular vergence ability decreased after viewing 3D movies with poorer stability of stereo-acuity and tear film. These findings (Iatsun et al., 2015; Cho et al., 2017) are thought to back up these theories about the origins of visual fatigue (Chen et al., 2015).

Park et al. (2015) underscored that viewing 3D content requires increased use of cognitive resources for information processing in contrast to 2D content. Furthermore, during 3D video viewing, brain fatigue can cause the degradation of the human visual system. Hence, Park et al. (2015) expressed visual fatigue as brain fatigue, indicating cumulative cognitive load. Moreover, Daniel and Kapoula (2019) analyzed visually normal young participants and consolidated the link between cognition and the high quality of single binocular vision. Furthermore, a recent review by Orru and Longo (2019) shows that visual input relates to working memory as well as auditory input, forming a major premise of the Cognitive Load Theory. Taken together, the association between increased visual fatigue and cognitive decline induced by 3D viewing is considered reasonable. However, it is unclear whether 3D viewing decreases overall cognitive function or merely cognitive function related to vision. If the study by Park et al. (2015) is correct, reduced performance is expected not only in a visual task but also in other cognitive tasks such as an auditory task after 3D viewing. Considering previous findings on the effects of 3D viewing on the visual processing, cognitive functions, and emotions of the brain (Yao et al., 2022), it is important to investigate brain activity during the cognitive task to understand the interaction between visual fatigue and cognitive functions.

Objective evaluation of the degree of visual fatigue has been performed using critical flicker fusion frequency (CFF) (Lin et al., 2017, 2021; Sugita et al., 2019). Functional near-infrared spectroscopy (fNIRS) is a non-invasive and portable neuroimaging modality used to measure oxygenated and deoxygenation hemoglobin in the brain (Pinti et al., 2015; Nogueira et al., 2022). Thus, the fNIRS device makes it possible to record brain activity during cognitive performance. This study examined the effects of visual fatigue induced by watching a 1-h movie on cognitive performance for the visual and auditory tasks. Furthermore, the processes involved in the reduction of cognition caused by 3D viewing were investigated based on brain activity data obtained by fNIRS during these tasks. This study aimed to test the following hypotheses:

(1) Watching a 1-h movie on television in 3D induces visual fatigue (a decrease in CFF), resulting in impaired cognitive performance both for the visual and auditory tasks.

(2) Impaired cognitive performance can be attributed to brain activity changes in specific regions determined using fNIRS after 3D viewing.

## Materials and methods

### Participants

The main outcomes of this study were indices of visual fatigue, cognitive function, and brain activity before and after watching a 1-h movie on television in both 2D and 3D. Hence, *a priori* sample size estimation was performed for a two-way repeated-measures analysis of variance (ANOVA) using the G*Power software package (version 3.1.9.4; Kiel University) before recruiting participants. The input parameters were as follows: Statistical test = ANOVA: Repeated measures, within-between interaction; Effect size: *f* = 0.25 (medium) (Cohen, 1988); α err prob = 0.05; Power (1 – β err prob) = 0.80; Number of groups = 2; Number of measurements = 2; Corr among rep measures = 0.50; Non-sphericity correction *e* = 1. As a result, the total sample size was calculated to be 34 (= 17 persons × 2 conditions). Considering the possibility of dropouts, this study recruited a slightly larger number of participants (20 young men). They were free of cardiovascular and eye diseases and used personal computers and smartphones regularly. Persons with clear vision or those who used contact lenses in their daily lives for correction were recruited as participants because the participants were required to use special glasses while watching the 1-h movie on television (please see the section “Watching the 1-h movie on television”). Two of the 20 participants withdrew from the experiment due to health problems. Hence, 18 young men completed the experiment [age: 22 ± 1 year, height: 171.1 ± 5.2 cm, body mass: 68.6 ± 6.8 kg; mean ± standard deviation (SD)]. The current study was approved by the institutional ethics committee (No. 21-015) and conducted according to the guidelines outlined in the Declaration of Helsinki. The participants were informed of the purpose and potential risks of the study and provided written informed consent before participation.

### Experimental procedures

**FIGURE 1:**
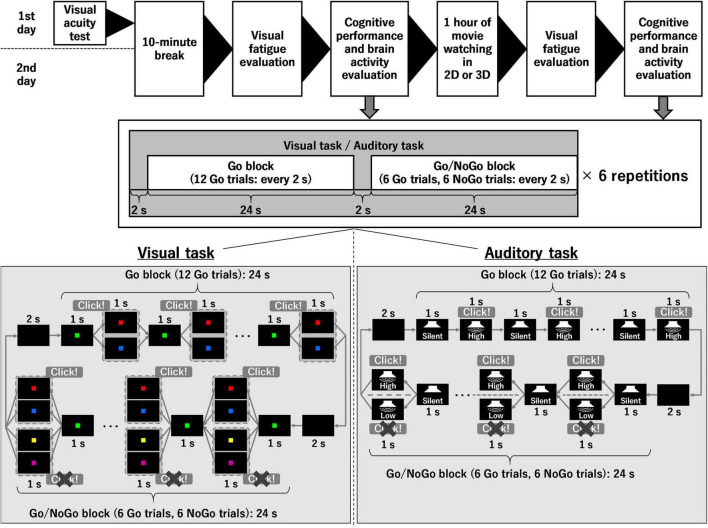
Experimental procedure and stimuli during cognitive tasks. 2D, two-dimension; 3D, three-dimension.

Figure 1 shows the experimental procedures of this study. Two conditions were used in the current study and were randomly performed for each participant on different days, namely, watching the 1-h movie on television in 2D (control condition) or 3D (3D condition). Before and after watching the 1-h movie, the degree of visual fatigue and cognitive performance were assessed. Brain activity during the evaluation of cognitive performance was also determined. Assessments of the degree of visual fatigue and cognitive performance were conducted within 30 min. Furthermore, the experiment was conducted in a quiet room with constant brightness.

On the first day, the visual acuity of the participants was confirmed as 1.0 or better in both eyes, either with the naked eye, or corrected with contact lenses, using a chart (height × width: 297 mm × 210 mm) with the Landolt rings posted 3 m in front of the participants. On both days, the participants were instructed to not consume caffeine from midnight on the day they entered the experiment to minimize the effect on the level of arousal. They were also instructed to keep their eyes open and not to gaze at anything for 10 min to assess the degree of visual fatigue before watching the 1-h movie, as their eyes required rest.

### Visual fatigue

In this study, CFF was determined using a digital flicker device (T.K.K.501c; Takei Scientific Instruments Co., Ltd., Niigata, Japan). The participants sat in a chair near a desk where the device was placed and peered at the device with both eyes. First, the frequency of the light blinking at 20 Hz was increased by 1 Hz per 0.5 s, and the frequency at which the participants felt the change in light from blinking to steady was measured. Second, the frequency of the light, which was at 60 Hz, was reduced by 1 Hz per 0.5 s, and the frequency at which the participants felt the light changed from steady to blinking was measured. In both cases, the frequency of the light started to change when the participants pressed and held the button. The button was then released when the participants judged that the light changed from blinking to steady, or from steady to blinking. The frequency of the light at that moment was recorded as CFF_*UP*_ (from blinking to steady) or CFF_*DOWN*_ (from steady to blinking). This was repeated three times, and the average value of CFF_*UP*_ and CFF_*DOWN*_ was evaluated.

### Cognitive performance and brain activity

Two cognitive tasks were performed in the present study, namely, a visual task and an auditory task. In both cognitive tasks, the combination of a Go block (12 Go trials) and a Go/NoGo block (6 Go trials and 6 NoGo trials) was repeated six times. In other words, each cognitive task included 108 Go trials and 36 NoGo trials; however, the participants were not informed that the cognitive task was divided into Go and Go/NoGo blocks, and they made Go/NoGo judgments in all 144 trials for each cognitive task. The combination of the Go block consisting of 12 Go trials and the Go/NoGo block consisting of 6 Go trials and 6 NoGo trials was drawn from the study of Herrmann et al. (2005). In both the Go block and the Go/NoGo block, there was an initial 2-s waiting period, followed by one trial every 2 s. That is, it took 52 s to perform one set of the Go block and the Go/NoGo block, and 312 s to complete the visual and auditory tasks, respectively. These tasks were controlled using PsychoPy 3 (installed on a personal computer in September 2021). Brain activity during the initial 2-s waiting period immediately before the Go/NoGo block was used as a baseline to determine the brain activity during the Go/NoGo block. As described in the Introduction, brain activity during the visual/auditory task was measured by fNIRS, in which a multi-channel fNIRS system (ETG-4000, Hitachi Medical Corporation, Tokyo, Japan) was equipped with 17 near-infrared light sources and 16 detectors was used. These sources and detectors were arrayed in a 3 × 11 lattice pattern and embedded in a soft silicone holder. The fNIRS system uses continuous wave laser diodes with two wavelengths (695 and 830 nm) as light sources. Optical fibers are used both for irradiation and for the detection of near-infrared light. The transmitted light was detected every 100 ms with avalanche photodiodes located 30 mm from the sources. The fNIRS system and the personal computer on which PsychoPy was installed were connected *via* a data logger (LabJack U3-HV, Sun System Supply Co., Ltd., Tokyo, Japan) to synchronize their data. During both the visual and auditory tasks, the brightness of the personal computer display and the volume of the personal computer speakers were set to maximum.

The participants sat on a chair and faced a personal computer screen placed on a desk. They were familiarized with two cognitive tasks (the visual and auditory tasks) for several minutes prior to starting the experiment. Subsequently, the soft silicon holder of the fNIRS system was placed on the participants’ foreheads. This configuration formed 52 measurement points. Afterward, the experiment was started. The order of the tasks, the visual task, and the auditory task, was randomly determined for each participant.

In the visual task (Figure 1), the participants clicked the space key of a personal computer with their right hand to start the task. The distance between the participant’s eye and the display was set to approximately 40 cm. A black screen was displayed for 2 s and then a green (RGB values: 127, 255, 0) square was shown for 1 s. In the Go/NoGo block, a red (RGB values: 255, 0, 0), blue (RGB values: 0, 0, 255), purple (RGB values: 128, 0, 128), or yellow (RGB values: 255, 255, 0) square was randomly superimposed for 1 s. The latter two colors (purple and yellow) were not used in the Go block. When the red or blue square was displayed, the participants clicked the space key of the personal computer as fast as possible (i.e., Go trial). When the purple or yellow square was displayed, the participants waited for the next question without doing anything (i.e., NoGo trial). In all cases, each colored square remained visible for 1 s. The green square was then displayed again for 1 s, followed by the two or four colored squares mentioned above for 1 s. After a total of 12 repetitions, the next block continued. Here, the size of the colored square was 25 mm × 25 mm.

The auditory task (see Figure 1) resembled the visual task until the task was initiated. When the participants pressed the space key to start the task, the black screen appeared and a 2-s wait period was allowed. Thereafter, each trial consisted of 1 s of silence followed by 1 s of sound. During the auditory task, the screen remained black and the participants were asked to keep their eyes open. In the Go/NoGo block, either high (2,000 Hz) or low (500 Hz) tones were randomly played. In the Go block, only the high tones were used. When the high tones were played, the participants clicked the space key of the personal computer as fast as possible. When the low tones were played, the participants waited for the next trial without doing anything. In both cases, the sound continued for 1 s. After a total of 12 repetitions, the next block continued.

In both visual and auditory tasks, reaction times in the Go trials were recorded. The reaction times that were recorded when the space key was pressed in the NoGo trials (i.e., incorrect trials) were not considered. Furthermore, the number of trials in which the space key was pressed in the Go trials and the number of trials in which the space key was not pressed in the NoGo trials were calculated.

### Watching the 1-h movie on television

Based on a previous study (Chen et al., 2015) investigating visual fatigue caused by watching 2D and 3D television, a 1-h viewing of an action movie [Transformers: The Last Knight (Blu-ray disc in Japanese)] was used as the fatigue task in the present study. The participants sat in a chair and wore headphones (TAPH805BK/10, Philips Japan Ltd., Tokyo, Japan) connected to a 3D-compatible television (KDL-40HX800, Sony Co., Ltd., Tokyo, Japan) with a DVD player (BDP-S6700, Sony Co., Ltd., Tokyo, Japan). The television was placed on a desk 1 m in front of the participants so that they could see the entire screen of the television. To induce intense eye movements, the participants’ neck was immobilized with a cervical collar (DR-127S, A-mi Global Co., Ltd., Busan, South Korea) during the movie viewing. When watching a movie in 3D, the participants wore 3D glasses (TDG-BR250, Sony Co., Ltd., Tokyo, Japan).

### Data and statistical analyses

For the data on CFF and cognitive performance (the response accuracy and the reaction time of the visual and auditory tasks), the Shapiro–Wilk test was used to assess the normality of the data. Hence, the data on CFF, the response accuracy of the visual and auditory tasks, and the reaction time of the auditory task were not normally distributed. Considering the present experimental design, two-way repeated-measures ANOVAs (i.e., parametric tests) were required. Therefore, the corresponding data were log-transformed before analyses as needed. For ease of interpretation, data in the text and figures are presented as means ± SD of raw data, unless noted otherwise.

The two-way repeated-measures ANOVA with two within-group factors [time (pre- and post-viewing), condition (control and 3D)] was used to evaluate changes in the data on CFF and cognitive performance (the response accuracy and the reaction time of the visual and auditory tasks) induced by the 1-h movie watching. When a significant interaction was detected, Bonferroni multiple comparisons were performed and an index of effect size from pretest-posttest-control group designs (*d*_*ppc*_) (Morris, 2008) was calculated. We determined that the changes in each parameter induced by the 1-h movie watching differed between the control and 3D conditions, when we performed the two-way repeated-measures ANOVA and found the following two things: (1) There was no difference in the values at pre-viewing between the conditions. (2) The time × condition interaction was confirmed. When the results of the two-way ANOVA are presented, η^2^ is also shown as an index of the effect size.

For the fNIRS data analyses, MATLAB (R2017a, The MathWorks Inc., Natick, MA, USA) and the plug-in-based analysis software [Open Platform of Transparent Analysis Tools for fNIRS (Open POTATo), National Institute of Advanced Industrial Science and Technology, Japan] (Sutoko et al., 2016) were used. First, the relative values of hemoglobin concentration changes (oxy-Hb signal changes) were preprocessed using Open POTATo with the following settings: filter function = butterworth (Dimension = 3), filter type = band-pass filter (high-pass filter = 0.02 Hz, low-pass filter = 0.8 Hz). If a motion artifact defined as a signal change larger than 0.1 mm over two successive samples (during 200 ms) was identified in a certain Go/NoGo block and the immediately following Go block, the data of the Go/NoGo block at that time were excluded. The availability of data from the last Go/NoGo block was determined based on the presence or absence of artifacts in that Go/NoGo block. Hence, the number of participants analyzed differed depending on the channel. Afterward, the average values of the oxy-Hb signal during a 16-s period starting from 8 s after the onset of the Go/NoGo block in each channel were calculated at pre-and post-viewing in the control and 3D conditions, respectively, for the visual and auditory tasks. These values were termed activation values and were used for further analyses.

To estimate the locations of the measurement channels in the Montreal Neurological Institute space, we used the coordinates data disclosed in a previous study (Sato et al., 2013) using the same channel arrangement with simultaneous measurements of fNIRS and functional magnetic resonance imaging. Additionally, a probabilistic registration method was used to generate 3D topographical maps (Singh et al., 2005). To detect channels in which brain activity was significantly higher in the Go/NoGo block compared to the baseline, the average values of the activation values at pre-viewing for the control and 3D conditions were calculated for each channel. Furthermore, a one-tailed one-sample *t*-test was used to determine whether the values were significantly higher than 0. Through these, we detected channels in which significant activation during the Go/NoGo block was observed. Although correcting the threshold of the multiple channel comparisons is desirable, the conventional correction method may possibly be too conservative and increase the risk of type 2 error in the 52-channel system. Hence, following previous studies that used fNIRS (Bae et al., 2017; Nakamichi et al., 2018), statistical significance was set at an uncorrected threshold of *P* < 0.05 when detecting the channels. Thereafter, to determine changes in the activation values induced by the 1-h movie watching in the detected channels, the two-way repeated-measures ANOVA with two within-group factors [time (pre- and post-viewing), condition (control and 3D)] was used. When a significant interaction was detected, Bonferroni multiple comparisons and the calculation of effect sizes were conducted, similar to the analyses of the CFF and cognitive performance data.

The two-way repeated-measures ANOVA and subsequent multiple comparisons were performed using SPSS (version 25.0, IBM, Armonk, NY, USA), the paired *t*-test was conducted using MATLAB (R2017a), and the effect sizes were calculated using Microsoft Excel (2016, Microsoft Corporation, Redmond, WA, USA). If two or more of CFF, cognitive performance, and brain activity showed significant changes before and after the 1-h viewing under the same conditions, repeated measures correlation was calculated using a web and standalone application for repeated measures correlation (rmcorrShiny) (Bakdash and Marusich, 2017; Marusich and Bakdash, 2021) to determine the overall within-individual relationship between them. Significance was set at *P* < 0.05. The absolute values of η^2^ or *d*_*ppc*_ were interpreted as η^2^ < 0.01 or *d*_*ppc*_ < 0.20 for trivial, 0.01 ≤ η^2^ < 0.06 or 0.20 ≤ *d*_*ppc*_ < 0.50 for small, 0.06 ≤ η^2^ < 0.14 or 0.50 ≤ *d*_*ppc*_ < 0.80 for medium, and 0.14 ≤ η^2^ or 0.80 ≤ *d*_*ppc*_ for large effects (Cohen, 1988).

## Results

### Visual fatigue

**FIGURE 2:**
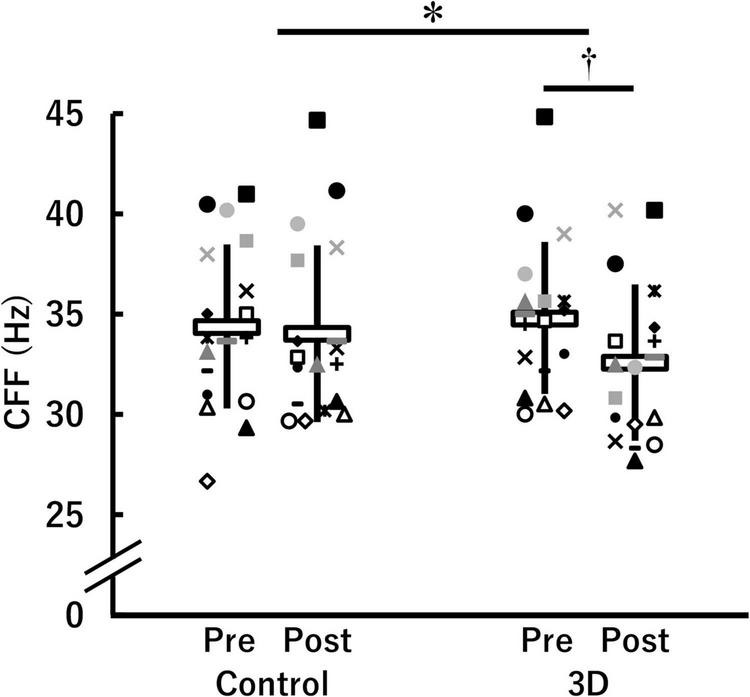
Critical flicker fusion frequency (CFF) before (Pre) and after (Post) watching a 1-h movie on television in 2D (Control) and 3D (*n* = 18). *Indicates a significant time (Pre, Post) × condition (Control, 3D) interaction (*P* < 0.05). ^†^Indicates a significant difference between Pre and Post in the same condition (*P* < 0.05). The data were not normally distributed under some conditions and thus log-transformed during the analysis. White horizontal bars and thick solid lines in the vertical direction indicate means ± standard deviation of raw data, respectively. Each symbol indicates individual data.

Figure 2 shows CFF in the control and 3D conditions at pre- and post-viewing. A significant time × condition interaction [*F*(1, 17) = 8.678, *P* = 0.009, η^2^ = 0.08 (medium)] was found. There were no significant differences between the conditions either at pre-viewing (control: 34.4 ± 4.1 Hz, 3D: 34.8 ± 3.8 Hz; *P* = 0.353) or post-viewing (control: 34.0 ± 4.4 Hz, 3D: 32.6 ± 3.9 Hz; *P* = 0.069). The task-induced change in CFF was significant in the 3D condition (pre-viewing > post-viewing; *P* < 0.001), but not in the control condition (*P* = 0.420). The absolute value of *d*_*ppc*_ was 0.48 (small). Thus, we observed that visual fatigue associated with watching the 1-h movie in 3D was greater.

### Cognitive performance

For the response accuracy of the visual task (control: 96.6 ± 3.9% at pre-viewing, 95.6 ± 4.5% at post-viewing; 3D: 95.7 ± 3.0% at pre-viewing, 95.1 ± 5.0% at post-viewing), a time × condition interaction [*F*(1, 17) = 0.184, *P* = 0.674, η^2^ < 0.01 (trivial)], a main effect of time [*F*(1, 17) = 2.560, *P* = 0.128, η^2^ = 0.04 (small)], and a main effect of condition [*F*(1, 17) = 1.033, *P* = 0.324, η^2^ = 0.03 (small)] were not significant. Similarly, for the response accuracy of the auditory task (control: 95.6 ± 6.4% at pre-viewing, 96.8 ± 3.8% at post-viewing; 3D: 98.3 ± 1.6% at pre-viewing, 96.4 ± 4.8% at post-viewing), neither the time × condition interaction [*F*(1, 17) = 4.366, *P* = 0.052, η^2^ = 0.06 (medium)] nor the main effects [time: *F*(1, 17) = 0.095, *P* = 0.762, η^2^ < 0.01 (trivial); condition: *F*(1, 17) = 1.826, *P* = 0.194, η^2^ = 0.03 (small)] were significant. These results indicated no significant difference in the effect of watching the 1-h movie in 2D and 3D on the response accuracy of the visual and auditory tasks.

**FIGURE 3:**
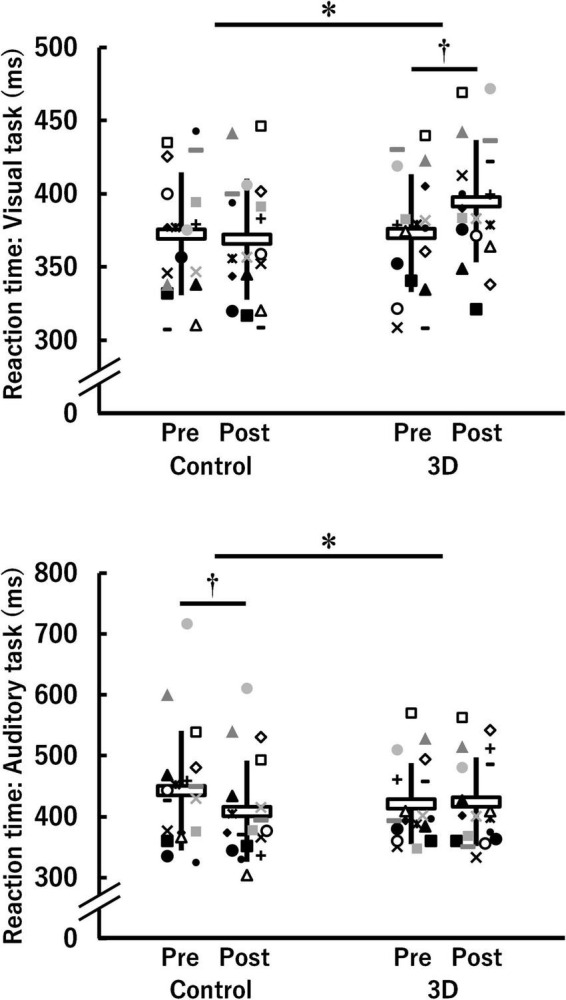
Reaction time for the visual and auditory tasks before (Pre) and after (Post) watching a 1-h movie on television in 2D (Control) and 3D (*n* = 18). *Indicates a significant time (Pre, Post) × condition (Control, 3D) interaction (*P* < 0.05). ^†^Indicates a significant difference between Pre and Post in the same condition (*P* < 0.05). The data were not normally distributed under some conditions and thus log-transformed during the analysis. White horizontal bars and thick solid lines in the vertical direction indicate means ± standard deviation of raw data, respectively. Each symbol indicates individual data.

Figure 3 shows the reaction time of both the visual and the auditory task in the control and 3D conditions at pre- and post-viewing. The reaction time of the visual task demonstrated a significant time × condition interaction [*F*(1, 17) = 5.216, *P* = 0.036, η^2^ = 0.06 (medium)]. A significant difference between the conditions was found at post-viewing (control: 368.9 ± 41.3 ms, 3D: 394.8 ± 41.8 ms; *P* = 0.009) but not at pre-viewing (control: 372.7 ± 42.1 ms, 3D: 373.0 ± 40.4 Hz; *P* = 0.974). The significant task-induced increase was found only in the 3D condition (3D: *P* = 0.027, control: *P* = 0.650). The absolute value of *d*_*ppc*_ was 0.61 [medium]. Thus, watching the 1-h movie in 3D worsened the reaction time of the visual task. For the reaction time of the auditory task, a time × condition interaction was significant [*F*(1, 17) = 9.016, *P* = 0.008, η^2^ = 0.06 (medium)]. Differences between the conditions were not significant either at pre-viewing (control: 443.2 ± 98.1 ms, 3D: 421.5 ± 66.2 ms; *P* = 0.228) or post-viewing (control: 408.6 ± 83.2 ms, 3D: 424.5 ± 72.5 ms; *P* = 0.273). The reaction time at post-viewing was significantly lower than that at pre-viewing only in the control condition (control: *P* = 0.003; 3D: *P* = 0.737). The absolute value of *d*_*ppc*_ was 0.46 (small). Thus, watching the 1-h movie in 2D rather than 3D resulted in faster reaction time of the auditory task.

### Brain activity in the Go/NoGo block

**FIGURE 4:**
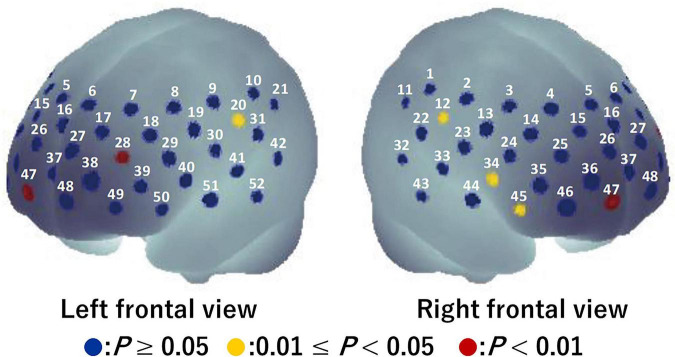
Activation map during Go/NoGo blocks in the visual task before watching a 1-h movie using a one-tailed one-sample *t*-test. Blue circles indicate *P* ≥ 0.05, yellow circles indicate 0.01 ≤ *P* < 0.05, and red circles indicate *P* < 0.01.

**FIGURE 5:**
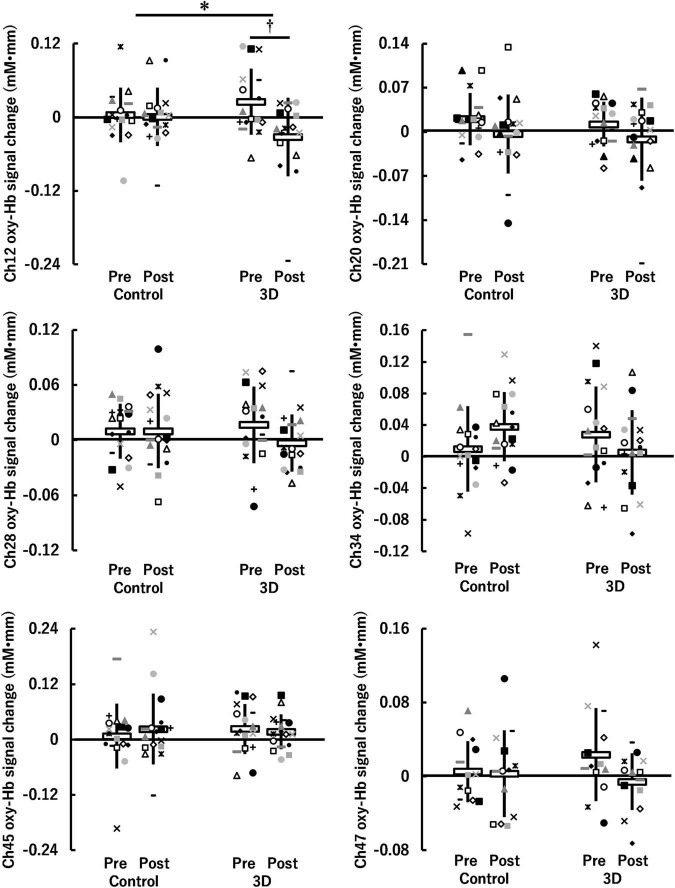
Hemoglobin concentration changes (oxy-Hb signal changes) during Go/NoGo blocks in the visual task before (Pre) and after (Post) watching a 1-h movie on television in 2D (Control) and 3D. *Indicates a significant time (Pre, Post) × condition (Control, 3D) interaction (*P* < 0.05). ^†^Indicates a significant difference between Pre and Post in the same condition (*P* < 0.05). White horizontal bars and thick solid lines in the vertical direction indicate means ± standard deviation, respectively. Each symbol indicates individual data (ch12: *n* = 16, ch20: *n* = 16, ch28: *n* = 17, ch34: *n* = 16, ch45: *n* = 17, ch47: *n* = 13).

For the visual task, significant activation during the Go/NoGo block was found in six channels (ch12, ch20, ch28, ch34, ch45, and ch47) (*P* = 0.008–0.043; Figure 4). The brain regions corresponding to the activation channels were estimated as follows: ch12 and ch20 are located around the right and left primary somatosensory cortex, respectively; ch28 is located around the left dorsolateral prefrontal cortex; ch34 and ch45 are located around the right inferior frontal gyrus; and ch47 is located around the right frontal pole. Supplementary Table 1 summarizes the statistical results of the two-way repeated measures ANOVA (*F*- and *P*-values and η^2^) on the detected channels. There was a significant time × condition interaction [*F*(1, 15) = 5.986, *P* = 0.027, η^2^ = 0.07] only in ch12 (see Figure 5). No significant time × condition interaction and main effects of time and condition were found in the other channels. In ch12, differences in the oxy-Hb signal change between the conditions either at pre-viewing (*P* = 0.329) or post-viewing (*P* = 0.071) were not significant, and the task-induced oxy-Hb signal change was greater at pre-viewing than at post-viewing only in the 3D condition (*P* = 0.011; control: *P* = 0.840). The absolute value of *d*_*ppc*_ in ch12 was 1.01 (large). These results indicated that brain activity during the Go/NoGo block in the visual task induced by watching the 1-h movie in 3D decreased only in ch12.

**FIGURE 6:**
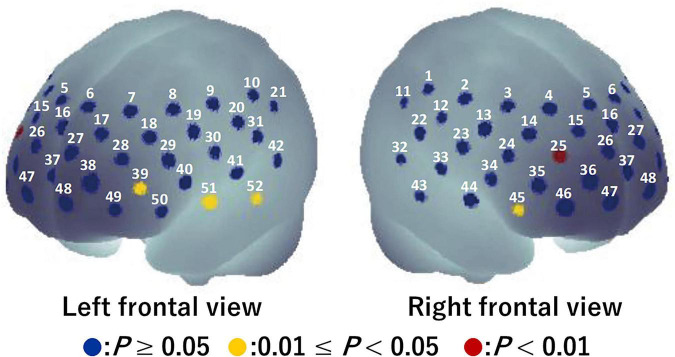
Activation map during Go/NoGo blocks in the auditory task before watching a 1-h movie using a one-tailed one-sample *t*-test. Blue circles indicate *P* ≥ 0.05, yellow circles indicate 0.01 ≤ *P* < 0.05, and red circles indicate *P* < 0.01.

**FIGURE 7:**
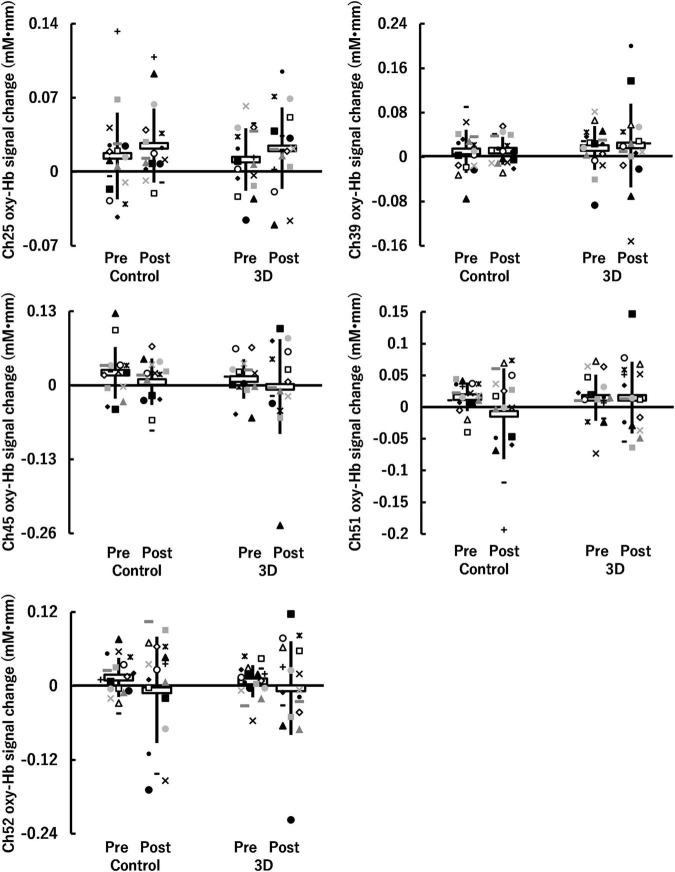
Hemoglobin concentration changes (oxy-Hb signal changes) during Go/NoGo blocks in the auditory task before (Pre) and after (Post) watching a 1-h movie on television in 2D (Control) and 3D. Neither a time (Pre, Post) × condition (Control, 3D) interaction nor main effects of time and condition were significant (*P* ≥ 0.05). White horizontal bars and thick solid lines in the vertical direction indicate means ± standard deviation, respectively. Each symbol indicates individual data (ch25: *n* = 17, ch39: *n* = 17, ch45: *n* = 15, ch51: *n* = 17, ch52: *n* = 18).

For the auditory task, five channels (ch25, ch39, ch45, ch51, and ch52) were detected in which brain activity was significantly higher in the Go/NoGo block compared to in the Go block (*P* = 0.010–0.024) (see Figure 6). The brain regions corresponding to the activation channels were as follows: ch25 and ch39 are located around the right and left dorsolateral prefrontal cortex, respectively; ch45 is located around the right inferior frontal gyrus; and ch51 and ch52 are located around the left middle temporal gyrus. Supplementary Table 2 summarizes the statistical results of the two-way repeated-measures ANOVA (*F*- and *P*-values and η^2^) on the detected channels. Among all channels, a time × condition interaction and main effects of time and condition were not significant (*P* = 0.131–0.951, η^2^ < 0.03; see Figure 7). Thus, no significant changes in brain activity during the Go/NoGo block in the auditory task were observed following the 1-h movie viewing in either the control or 3D conditions.

### Repeated measures correlations

**FIGURE 8:**
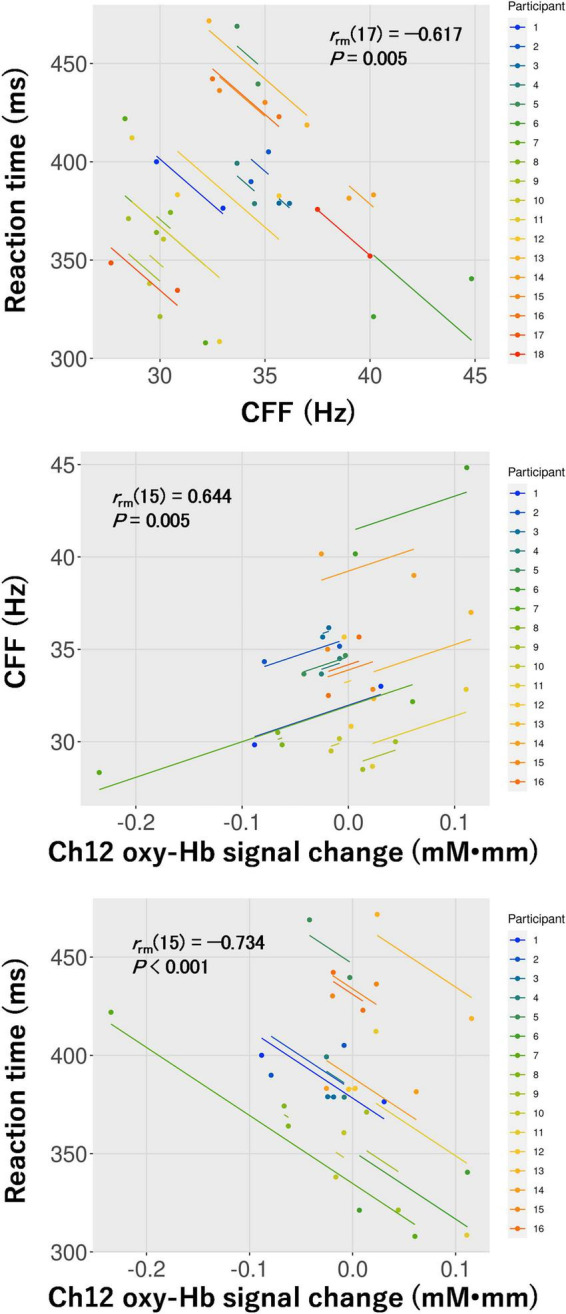
Repeated measures correlations between critical flicker fusion frequency (CFF) and the reaction time for the visual task (top: *n* = 18), CFF and hemoglobin concentration changes (oxy-Hb signal change) in ch12 (middle: *n* = 16), and the reaction time for the visual task and oxy-Hb signal change in ch12 (bottom: *n* = 16) in the 3D condition.

Based on the results of the two-way repeated-measures ANOVA, repeated measures correlations among CFF, the reaction time for the visual task, and the oxy-Hb signal change in ch12 for the visual task were calculated in the 3D condition. Subsequently, the repeated measures correlations among CFF and the reaction time [*r*_*rm*_ (17) = –0.617, *P* = 0.005], CFF and the oxy-Hb signal change [*r*_*rm*_ (15) = 0.644, *P* = 0.005], and the reaction time and the oxy-Hb signal change [*r*_*rm*_ (15) = –0.734, *P* < 0.001] were all significant (Figure 8).

## Discussion

In this study, visual fatigue induced by watching the 1-h movie on television was greater in the 3D condition than in the control condition. Furthermore, the reaction time for the visual task deteriorated after watching the 1-h movie on television in 3D. These results, together with the finding of the significant repeated measures correlation between CFF (i.e., visual fatigue) and the reaction time for the visual task, suggest that visual fatigue induced by watching a 1-h movie on television in 3D results in impaired cognitive performance only in the visual task. After watching the 1-h movie on television in 3D, decreased activity around the right primary somatosensory cortex (ch12) during the Go/NoGo block was observed. Furthermore, the study observed decreased cognitive performance in the visual task based on the close relationship between them. Thus, the decreasing brain activity around the right primary somatosensory cortex after watching the movie in 3D reduced cognitive performance in the visual task. This study hypothesized that (1) watching a 1-h movie on television in 3D induces visual fatigue (a decrease in CFF), resulting in impaired cognitive performance for both the visual and auditory tasks, and (2) this impaired cognitive performance can be attributed to brain activity changes in specific regions after 3D watching. The results of this study reject the first hypothesis and support the second.

Visual fatigue was evaluated using CFF in this study, consistent with previous studies (Lin et al., 2017, 2021; Sugita et al., 2019). In contrast to the control condition, the decrease in CFF was pronounced in the 3D condition (see Figure 2), indicating that visual fatigue occurred in the 3D condition. This result is consistent with a study by Sugita et al. (2019), which contrasted the effect of visual fatigue induced by watching a 10-min movie in both 2D and 3D. As described in the Introduction, Iatsun et al. (2015) reported that the number of saccades, fixations, and fixation duration decreased with time during 3D viewing. However, no changes in these parameters were observed over time during 2D viewing. Cho et al. (2017) reported that accommodative response and ocular movements revealed noticeable changes after 3D viewing as opposed to 2D viewing. Furthermore, the ability for both the accommodation and binocular vergence decreased after viewing 3D movies with poorer stability of stereo-acuity and tear film. Therefore, it was expected that the 1-h movie viewing in the 3D condition caused greater visual fatigue than in the control condition in this study.

Regarding cognitive performance, the reaction time for the visual task was significantly longer after watching the movie in 3D but not in 2D (see Figure 3). However, no differences were observed in the response accuracy of the visual task between pre- and post-viewing and the control and 3D conditions. Consistent with a previous study, this study found that cognitive performance on the visual task was significantly impaired in 3D (Park et al., 2015). Additionally, a significant repeated measures correlation between CFF and the corresponding reaction time in the 3D condition was found (see Figure 8). Delays in transmission of visual information measured using electroencephalography served as an appropriate measure for visual fatigue (Lambooij et al., 2009; Iatsun et al., 2015), indicating that increased visual fatigue results in slower visual information processing in the brain. Thus, the results demonstrating that increased visual fatigue induced by watching the movie in 3D leads to impaired cognitive performance on the visual task are justified.

The Go/NoGo task requires executive function, including selective attention, response inhibition, and interference control (Diamond, 2013). The activation during the Go/NoGo block included channels located around the dorsolateral prefrontal cortex for both the visual task (ch28) and the auditory task (ch25 and ch39), which is consistent with the results of a previous visual task study (Herrmann et al., 2005). Additionally, the results of the corresponding activation around the inferior frontal gyrus for both tasks (ch45) are in line with those of another previous study (Steele et al., 2013).

In ch12 of the six channels detected in the visual task, different changes in brain activity during the Go/NoGo block induced by watching the movie were observed between the control and 3D conditions (see Figure 5). Thus, brain activity around the right primary somatosensory cortex was lower during the Go/NoGo block in the visual task at post-viewing compared to pre-viewing in the 3D condition. Furthermore, the repeated measures correlation between the reaction time and the oxy-Hb signal change around the right primary somatosensory cortex for the visual task was significant in the 3D condition [*r*_*rm*_ (15) = –0.734]. These results indicate that cognitive performance was lower in participants with less brain activity. Moreover, there was a significant repeated measures correlation between CFF and the oxy-Hb signal change around the right primary somatosensory cortex for the visual task in the 3D condition [*r*_*rm*_ (15) = 0.644], indicating that brain activity around the right primary somatosensory cortex in the visual task deteriorated among the participants with increased visual fatigue after watching the 1-h movie in 3D. It has been suggested that the lateral geniculate nucleus of the thalamus receives visual information from the eye (Bundesen et al., 2005; Pregowska et al., 2019) and that the thalamus contributes to the conscious perception or awareness of visual information (Saalmann and Kastner, 2011). Furthermore, the thalamus connects to the primary somatosensory cortex structurally and functionally; thus, the interactions between them are pertinent to the cognitive process (Kang et al., 2018). High-demand cognitive processes require greater brain activity and greater oxygen supply (Park et al., 2015). Thus, our results suggest that failures or delays in transmitting visual information to the thalamus, and thus to the primary somatosensory cortex associated with visual fatigue, affected the decrease in brain activity around the primary somatosensory cortex, which led to poor cognitive performance in the visual task.

For the auditory task, there was a significant time × condition interaction (see Figure 3) in the reaction time, indicating that cognitive performance improved after viewing the 1-h movie in the control condition; however, it did not change in the 3D condition. Considering the inverted-U relationship between arousal level and performance (Schmidt and Lee, 2000), eye and brain stimulation caused by viewing the 1-h movie in the control condition might have increased arousal levels. In contrast, two possibilities were considered for the 3D condition. First, the decreased cognitive performance due to increased visual fatigue offsets the increased cognitive performance due to increased arousal levels. Second, eye and brain stimulation induced by the 1-h movie viewing in the 3D condition was relatively strong in comparison to the control condition, resulting in reduced arousal levels in the 3D condition. Owing to the lack of variation in the brain activity during Go/NoGo blocks in the auditory task between the conditions (see Figure 7), the latter possibility seems more justified. Thus, it is unlikely that visual fatigue significantly affected cognitive performance in the auditory task. Considering this simultaneously with the results of cognitive performance and brain activity in the visual task, it can be assumed that watching a movie in 3D can affect cognitive performance when working on a cognitive task that requires vision, without affecting the cognitive function itself.

The importance of 3D applications in our daily lives will continue to grow. Therefore, it is essential to mitigate challenges associated with the use of 3D applications that can threaten human health. This study revealed that increased visual fatigue results in failures or delays in transmitting visual information to the brain, thus impairing cognitive performance for visually demanding tasks. This suggests that performing tasks that require visual information, such as running in the dark or driving a car, immediately after using a 3D application, may create unexpected risks in our lives. Thus, the findings of this study will be useful in outlining precautions for the use of 3D applications.

This study has several limitations. First, the experiment was conducted using a specific 2D/3D display (television) released over 10 years ago to compare 2D and 3D viewing under relatively close conditions. As mentioned in the paragraph above, a variety of 3D applications are in the process of development. Therefore, the visual fatigue prompted by the use of modern 3D applications may be lower than the visual fatigue obtained in this study. Second, the fNIRS data was a limiting factor because this study could not remove the effect of extracranial hemodynamic changes such as skin blood flow due to the limitation of system configuration. However, this study observed less motor load in the Go/NoGo block period than in the Go block (control condition). Therefore, it is unlikely that the activation signal was due to systemic hemodynamic changes. Third, only executive function was assessed in this study. Investigating the effects of 3D movie viewing-induced visual fatigue on other aspects of cognitive function should also be performed in future research to further strengthen the findings of this study. Given that the effect of visual fatigue on brain activity was observed in the brain regions related to vision, but not in the brain regions associated with executive function, it is expected that similar results may be obtained for any cognitive task that requires vision. Last, when detecting the channels in which brain activity was significantly higher in the Go/NoGo block than the baseline, statistical significance was set at an uncorrected threshold of *P* < 0.05, consistent with previous studies that used fNIRS (Bae et al., 2017; Nakamichi et al., 2018). This method was adopted because the statistical results are not overly conservative and type 2 errors are not too high. However, the risk of type 1 errors existed. In such cases, it will be critical to establish an appropriate method that balances the risk of type 1 and type 2 errors in the future.

In conclusion, this study examined the effect of visual fatigue induced by watching a 1-h movie on television in 3D on cognitive performance in the visual and auditory tasks. In addition, the processes involved in the cognitive performance reduction caused by 3D viewing were investigated based on brain activity data obtained by fNIRS during these tasks. The reaction time for the visual task deteriorated after watching the 1-h movie on television in 3D. Furthermore, there was a significant repeated measures correlation between visual fatigue and the reaction time for the visual task (i.e., the reaction time was longer for those with greater visual fatigue) in the 3D condition. Moreover, reduced activity around the right primary somatosensory cortex was observed during the Go/NoGo block in the visual task, and the repeated measures correlations of this brain activity with visual fatigue as well as the reaction time for the visual task were both significant (i.e., visual fatigue was greater and the reaction time was longer in participants with lower brain activity) in the 3D condition. These results suggest that failures or delays in transmitting visual information to the thalamus, and thus to the primary somatosensory cortex due to visual fatigue induced by watching a movie in 3D affected the reduction in brain activity around the primary somatosensory cortex, resulting in poor cognitive performance in the visual task.

## Data availability statement

The raw data supporting the conclusions of this article will be made available by the authors, without undue reservation.

## Ethics statement

The studies involving human participants were reviewed and approved by the Ethics Committee of the Shibaura Institute of Technology. The patients/participants provided their written informed consent to participate in this study.

## Author contributions

RA, HS, KH, MK, and SA conceived and designed the research. TH and MK conducted the experiments. RA, HS, and TH analyzed the data. RA and HS drafted the manuscript and prepared the figures. All authors interpreted the results of the research, edited, critically revised, and approved the final version of the manuscript.
